# The Question of Economics

**Published:** 1917-09-15

**Authors:** 


### NURSING AFFAIRS IN IRELAND.

### The Question of Economics.

I pointed out in a previous letter * that the economic problem is that which most seriously concerns the Irish nurse. I propose, with your permission, to discuss this subject, which is somewhat difficult as well as somewhat delicate, and which, probably on this account, is usually shelved.

### Why the Nursing Profession is Chosen.

Why does a woman become a nurse? This elementary question bears on this issue, and the answer ought to be free from obscurity or doubt. Is she, according to her motives and her ambition, out for a career simply and only, or is her object in life partially or wholly philanthropic? I am not aware that this question has ever been seriously raised. Nevertheless, it is a serious question, for one constantly finds newspaper and other references to the nobility of nursing, the self-sacrifice that it entails, the patience and skill that it demands, and yet, on the other hand, in Ireland at least, there is no class of worker so ill-paid. As these facts are in conflict, it becomes necessary to settle definitely at the outset the object in view of a young woman who enters a hospital as a probationer. If this is partially or entirely philanthropic, and the reward a secondary consideration or altogether of negligible importance, there is no more to be said. But I venture to suggest that the candidate-probationer cherishes hopes and ambitions altogether different. Having considered a variety of occupations, and her own fitness for any of them, she makes up her mind that, taking all the circumstances into consideration, nursing is for her the most suitable, and the more remunerative her particular work proves ultimately to be the better she is pleased. Other girls take to medicine, to secretarial work, to millinery; but the common desire of all of them is to achieve success and the universally recognised reward of such success, a sufficiently large income to provide for the necessities of life while she is a worker, as well as when age or infirmity compels her to retire.

### The Hospital a Bird of Prey?

I have referred to this as a somewhat delicate discussion, and for the reason that it calls for critical remarks concerning hospitals. It is only with reluctance and hesitancy that anybody can attempt to condemn hospital policy. These institutions are for the benefit of the sick poor, and they confer incalculable benefit on the inhabitants of crowded neighbourhoods where the struggle for life is extreme. They are, in the main, supported by voluntary contributions and by legacies from people whose motives are without alloy. If a hospital were to be removed or blotted out in such places the result would be calamitous to a degree of which well-off people can have no adequate conception. Medical men attend without fee, but it cannot be denied that in so doing they receive as well as give. In giving of their time and talent to the suffering poor they find benefit to themselves. Now while all this, and much more beside, is attributable to the hospital, one cannot resist the conclusion that so far as the nurse is concerned the hospital is very often to her a bird of prey. There are, doubtless, notable exceptions, but it is not possible to arrive at general and accurate conclusions by taking account only of exceptional standards.

### Private Nursing Staff Finances.

To be specific, and to come to figures, I have before me the annual reports of various Dublin hospitals. At random I select that of the Royal City of Dublin, probably second to none for efficiency, popularity, and dimensions. The report is not a private document, for on the front page appears the message, "It is earnestly requested that this report may be circulated." The regulations for probationers provide that they must serve the hospital for a period of four and a half years, and that they must pay an entrance fee of thirty-five guineas. During these years they are paid as follows: First year, £3; second year, £3; third year, £12; fourth year, £18; last half-year, £10. The first three years are for training. Without further comment I think it is fair to assume that when the probationer has completed her period of service she is in no respect in debt to the hospital. Now, turning to the balance-sheet, I find in the receipts the following entry:

| | |
|---|---|
| Nursing Institution— | |
| Private fees ... ... ... | £718 18 0 |
| Nurses' and probationers' fees ... | 627 1 8 |
| | £1,345 19 8 |

And under the heading "Expenditure" appears the entry: Salaries—Staff sisters and nurses, £787 4s. 10d. It is not specified whether the private fees include sums paid by patients who are maintained in the hospital. But, allowing for this, it is evident that a large proportion of the total amount is derived from the services of nurses supplied to private residences, as this constitutes a prominent feature of the hospital's work. From these figures it appears that the hospital, so far as nursing is concerned, is run at a considerable profit. The moneys earned by the nurses are utilised in the cause of charity. The probationers work for nothing for a period of four years, and they are not eligible to enter until they have passed the age of twenty-one. I have looked in vain in the balance-sheet for some entry to provide pensions for the nurses.

### The Nurse Bereft of Her Earnings.

I leave these figures for the consideration of your readers, and I repeat the question with which I began this letter—Is a nurse a professional philanthropist, or is she a woman pursuing a career with the object of earning a competence? However much one may regret to have to question the domestic policy of institutions of which we are all proud, the issue is unavoidable. The nurse is, with the best of intentions, bereft of her earnings. They are taken from her, willy-nilly, and given to the poor. Hence my suggestion that the supreme consideration of the Irish nurse is the economic problem. Will the College of Nursing take the bold step of endeavouring to find a solution?

IERNE.

* See The Hospital, September 1, p. 444.

